# Prognostic influence of endoscopic ultrasound-guided fine needle aspiration in IPMN-derived invasive adenocarcinoma

**DOI:** 10.1186/s12885-018-4896-2

**Published:** 2018-10-12

**Authors:** Rei Suzuki, Hiroki Irie, Tadayuki Takagi, Mitsuru Sugimoto, Naoki Konno, Yuki Sato, Ko Watanabe, Jun Nakamura, Shigeru Marubashi, Takuto Hikichi, Hiromasa Ohira

**Affiliations:** 1 Department of Gastroenterology, Fukushima Medical University School of Medicine, 1 Hikarigaoka, Fukushima, 960-1295 Japan; 2 Department of Endoscopy, Fukushima Medical University Hospital, Fukushima, Japan; 3 Department of Hepato-Biliary-Pancreatic and Transplant Surgery, Fukushima Medical University School of Medicine, Fukushima, Japan

**Keywords:** EUS-FNA, IPMN, Adenocarcinoma, Prognosis

## Abstract

**Background:**

Endoscopic ultrasound-guided fine needle aspiration (EUS-FNA) for mucinous cystic neoplasm of the pancreas carries a potential risk of inducing peritoneal tumor cell dissemination. We investigated the diagnostic yield and safety of EUS-FNA-based cytology of cells obtained from the pancreatic invasion site of intraductal papillary-mucinous neoplasm-derived adenocarcinoma (IPMC).

**Methods:**

We included 22 surgically resected IPMCs and 84 pancreatic ductal adenocarcinomas (PDACs). Among the IPMC cases, 14 did not undergo EUS-FNA before surgical resection. The diagnostic yield of EUS-FNA was compared between IPMC and PDAC. Additionally, prognosis (relapse-free and overall survival time after resection) and the rate of peritoneal dissemination were compared among IPMC with EUS-FNA, IPMC without EUS-FNA, and PDAC. A survival analysis was performed using the Kaplan-Meier method and log-rank test.

**Results:**

(EUS-FNA diagnosis) There were no significant differences in the number of needle passages (PDAC 2.5 vs. IPMC 2.0 passages, *P* = 0.84) or puncture route (stomach/duodenum: 2/6 vs. 45/39, *P* = 0.29). However, the correct diagnosis rate was significantly higher in PDAC (92.9%) than in IPMC (62.5%) (*P* = 0.03). No procedure-related adverse events occurred. Peritoneal lavage cytology performed during the operation was negative in all cases. (Prognosis) Among IPMC with EUS-FNA, IPMC without EUS-FNA, and PDAC, there were no significant differences in relapse-free survival (21.0 vs. 22.4 vs. 12.5 months, respectively; *P* = 0.64) or overall survival time (35.5 vs. 53.1 vs. 35.9 months, respectively; *P* = 0.42), and peritoneal dissemination was detected during the observation period in 25%, 28.5%, and 21.4% cases, respectively (*P* = 0.82).

**Conclusion:**

Even though a correct diagnosis was more difficult to obtain in IPMC than in PDAC, IPMC allows specimens to be obtained without influencing the rate of recurrence and prognosis.

## Background

Intraductal papillary mucinous neoplasm (IPMN) constitutes a broad pathological spectrum: hyperplasia (benign), low-grade dysplasia (adenoma), high-grade dysplasia (carcinoma in situ), and adenocarcinoma [1]. Even though 2 guidelines have proposed the use of several key imaging features (e.g., mural nodule, dilated pancreatic duct) for risk stratification of malignancy, the diagnostic yield of these criteria requires further improvement [2–4]. Therefore, a considerable number of studies utilizing imaging studies, cytology, and cystic fluid analysis (for tumor markers, molecular markers, etc.) have attempted risk stratification in IPMN for appropriate management [5–7]. Among these methods, cytology is one of the most important factors for differentiating IPMNs and can affect patient management. In Japan, endoscopic retrograde cholangiopancreatography (ERCP) is widely accepted as a technique for obtaining specimens from IPMNs for cytology. However, as we previously reported in a meta-analysis of 13 international studies with 483 IPMN patients, cytology of specimens obtained from ERCP showed good specificity but poor sensitivity in distinguishing benign from malignant IPMNs. The pooled sensitivity was 35.1%, while specificity was 97.2% [8]. In western countries, EUS-FNA is a standard technique used to obtain cystic fluid from IPMNs that has better sensitivity (64.8%) than is obtained using ERCP-based cytology; however, this procedure carries a potential risk of tumor cell dissemination and is considered contraindication in Japan [9].

To maximize the diagnostic yield of EUS-FNA in pancreatic lesions, we performed EUS-FNA at the pancreatic invasion site of IPMN-derived adenocarcinomas (IPMCs) while avoiding puncturing the cystic component of the disease. In the current study, we aimed to determine the diagnostic yield and prognostic influence of EUS-FNA used in IPMC in our cases.

## Methods

### Objectives

This retrospective study evaluated data obtained from 106 consecutive patients (22 IPMC and 84 PDAC) who underwent surgical resection at Fukushima Medical University Hospital between April 2006 and June 2016. Before EUS-FNA was performed, written informed consent was obtained from all patients. The study protocol conformed to the ethical guidelines of the 1975 Declaration of Helsinki and was approved by the institutional review committee of Fukushima Medical University. Regarding EUS-FNA for IPMC, we selected cases in which we detected signs of pancreatic parenchymal invasion in EUS and secured a safe route to avoid puncturing the cystic component of the tumor.

The exclusion criteria were one or more of the following: (1) IPMN without any sign of pancreatic parenchymal invasion, (2) postoperative follow-up duration < 30 days, (3) absence of cross-sectional imaging reports (computed tomography [CT] or magnetic resonance imaging [MRI]) during follow-up and (4) preoperative chemo-radiation therapy.

### FNA technique

**Fig. 1:**
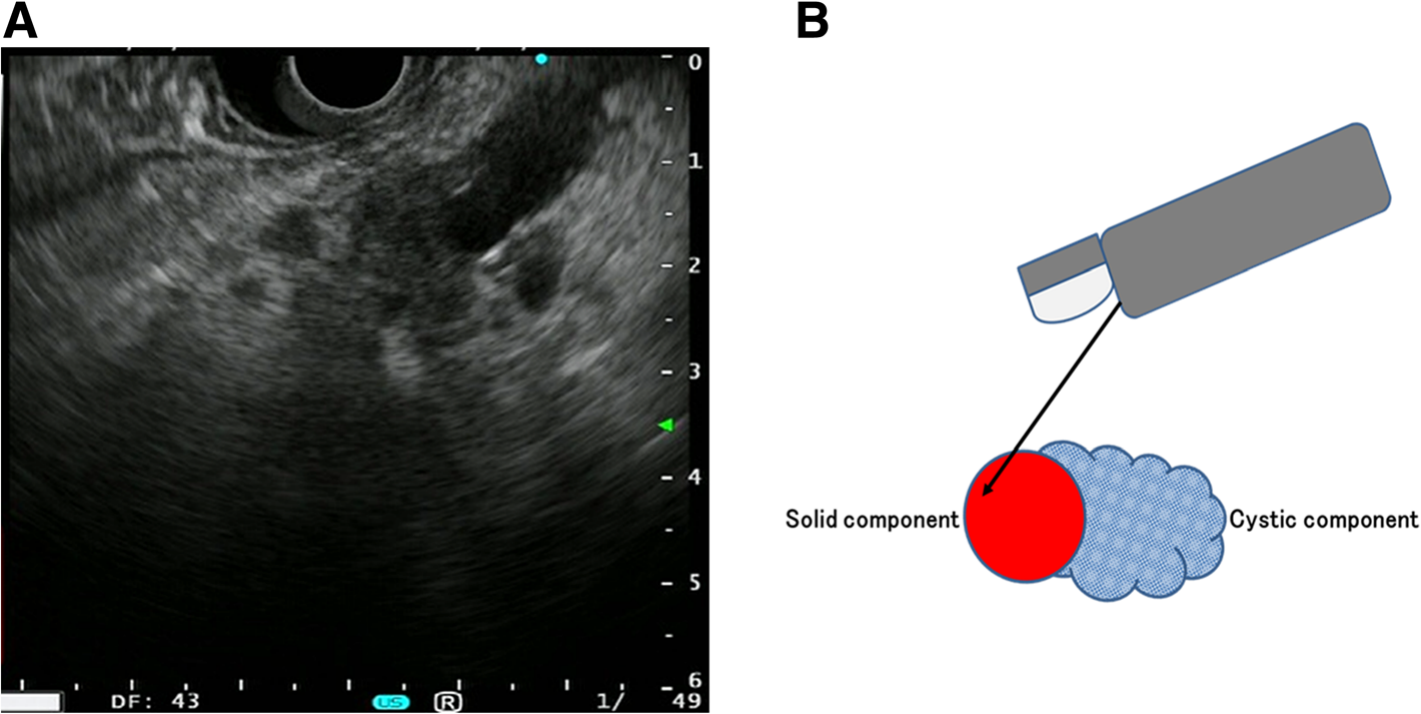
EUS-FNA for IPMC. **a** EUS detected cystic lesion with invasion of the pancreatic parenchyma. **b** Schematic illustration of EUS-FNA for IPMC with pancreatic invasion

EUS-FNA was performed using a curved linear-array echoendoscope (GF-UCT240/260, GF-UC240P; Olympus Medical Systems Corp., Tokyo, Japan) in conjunction with EU-ME1 or EU-ME2 (Olympus Medical Systems Corp., Tokyo, Japan) and Expect (Boston Scientific Corp., Natick, USA) or Echotip (Cook Medical) 22- and 25-gauge FNA needles. EUS-FNA procedures were performed with moderate intravenous sedation using both midazolam and pentazocine. The needle was passed through the accessory channel of the echoendoscope and advanced through the GI wall under EUS guidance into the target lesion with visualization of the needle in real time to avoid the blood vessel and cystic components of the IPMC (Fig. 1a and b). After the echoendoscope was guided into the target lesion, the stylet was removed, and the needle was moved back and forth 10 times within the mass while suction was applied using a 10 mL syringe. The procedure was continued until the cytopathologist indicated an adequate amount of cells had been obtained. If the aspirate revealed only inflammatory or benign cells, several passes were made in different directions within the mass to minimize the sampling error of malignant lesions.

### Cytological evaluation

The cytological criteria used to report EUS-FNA results were based on the guidelines of the Papanicolaou Society of Cytopathology for fine needle aspiration and reporting [10]. We regarded Class I-II as benign, Class III as atypical/indeterminate, and Class I*V*/V as malignant.

### Variables

Clinical characteristics, including age, sex, tumor size, tumor location, T- and N- stage (based on the UICC classification, ver. 7), and serum levels of tumor markers (carcinoembryonic antigen [CEA] and cancer antigen 19–9 [CA19–9]) were compared between IPMC and PDAC. Regarding the diagnostic yield of EUS-FNA, the number of needle passages, puncture route (stomach or duodenum), correct diagnosis rate and rate of adverse events were evaluated. Relapse-free survival (RFS) was defined as the survival period during which patients survived after surgical resection with no signs of recurrence, and overall survival (OS) was defined as the time from treatment initiation to death from any cause. Cross-sectional image findings were used to detect recurrence.

### Statistics

Continuous variables were reported as the median (range). For categorical data, the chi-squared test or Fisher’s exact test was performed, as appropriate. To compare continuous variables, the Wilcoxon rank-sum test was performed. Median RFS and OS after surgical resection were calculated using the Kaplan-Meier method. All statistical analyses were performed with GraphPad Prism 7.0 (GraphPad, San Diego, CA, USA). *P* < 0.05 was considered statistically significant.

## Results

### Diagnostic yield of EUS-FNA in IPMC and PDAC

As shown in Table 1, we included 8 IPMC and 84 PDAC in the first analysis. Regarding diagnostic yield, there were no significant differences in the number of needle passages or the puncture route. However, the correct diagnosis rate was significantly lower in IPMC than PDAC (62.5% vs. 92.9%, *P* = 0.03) (Table 2). No adverse events were observed after EUS-FNA and peritoneal seeding in peritoneal lavage cytology during the operation.

**Table 1:** Clinical characteristics of patients

	IPMC with EUS-FNA (*n* = 8)	PDAC (*n* = 84)	IPMC without EUS-FNA (*n* = 14)	*P*
Age	71.0 (62.0–79.0)	71.5 (48.0–86.0)	72.0 (52.0–80.0)	0.89
Sex (M:F)	5:3	49:35	10:4	0.55
T-stage^a^ (T1-T2/T3-T4)	3/5	19/65	10/4	0.001
N-stage^a^ (N0/N1)	10/1	55/29	10/4	0.22

^a^JPS classification of pancreatic cancer ver.7 was applied

*EUS-FNA* Endoscopic ultrasound-guided fine needle aspiration, *IPMC* Intraductal papillary mucinous neoplasm-derived adenocarcinoma, *PDAC* Pancreatic ductal adenocarcinoma. *M* Male, *F* Female. Data was shown in median (range)

**Table 2:** EUS-FNA diagnosis

	IPMC (*n* = 8)	PDAC (*n* = 84)	*P*
Needle passes	3.0 (1.0–5.0)	2.0 (1.0–11.0)	0.33
Puncture route (stomach:duodenum)	6:2	44:40	0.27
Correct diagnosis (sensitivity)	62.5% (5/8)	92.9% (78/84)	0.03
Adverse events	0	0	1.00

*EUS-FNA* Endoscopic ultrasound-guided fine needle aspiration, *IPMC* Intraductal papillary mucinous neoplasm-derived adenocarcinoma, *PDAC* Pancreatic ductal adenocarcinoma

Data was shown in median (range)

**Table 3:** Pattern of recurrence after surgical resection

	IPMC with EUS-FNA (*n* = 8)	PDAC (*n* = 84)	IPMC without EUS-FNA (*n* = 14)	*P*
Total recurrence, no (%)	6 (75.0)	47 (55.9)	9 (64.3)	0.51
Local recurrence, no (%)	3 (37.5)	15 (17.8)	6 (42.8)	0.06
Liver or Lung metastasis, no (%)	2 (25.0)	24 (28.5)	3 (21.4)	0.85
Lymph node metastasis, no (%)	3 (37.5)	9 (10.7)	1 (7)	0.07
Peritoneal dissemination, no (%)	2 (25.0)	18 (21.4)	4 (28.5)	0.82

*EUS-FNA* Endoscopic ultrasound-guided fine needle aspiration, *IPMC* Intraductal papillary mucinous neoplasm-derived adenocarcinoma, *PDAC* Pancreatic ductal adenocarcinoma

### Prognostic influence of EUS-FNA for IPMC and PDAC

**Fig. 2:**
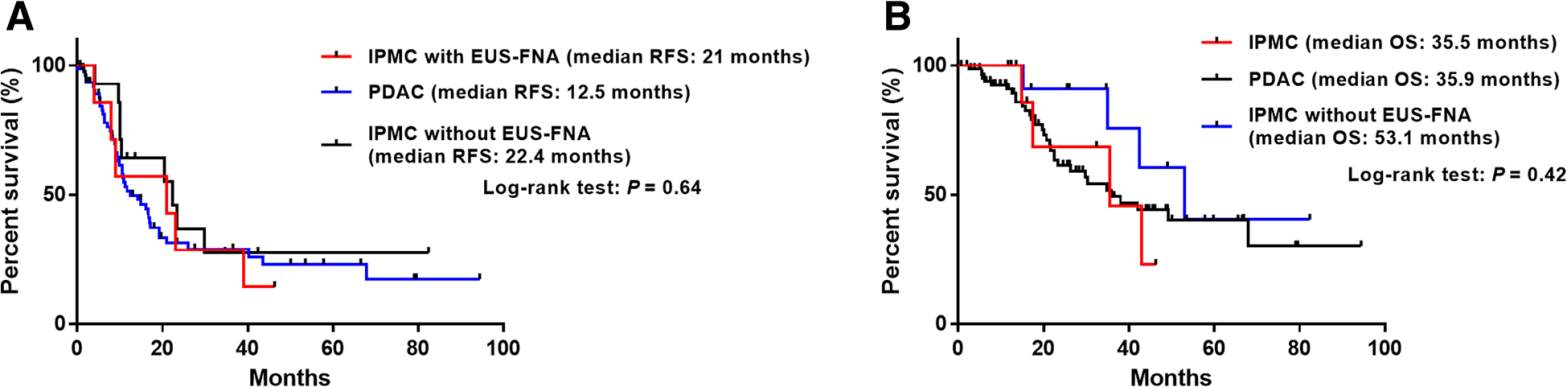
Survival analysis. There were no differences in (**a**) relapse-free survival (RFS) and (**b**) overall survival (OS) among the three groups

To clarify the prognostic influence of EUS-FNA for IPMC, we included 8 IPMC with EUS-FNA, 14 IPMC without EUS-FNA and 84 PDAC as controls. With regard for baseline clinical characteristics, there were no significant differences among the groups in age, sex and TNM N-stage (Table 1). Regarding T-stage, we observed a statistically significant difference among the 3 groups (*P* = 0.001). There were more patients with T1–2 tumors in IPMC without EUS-FNA (10 out of 14) than in PDAC (19 out of 84) (*P* = 0.0002). However, there was no significant difference in the T-stages of the tumors between IPMC with EUS-FNA (3 out of 8) and IPMC without EUS-FNA (*P* = 0.18) or PDAC (*P* = 0.34). In the survival analysis, there were no significant differences among the 3 groups in median RFS (21 months in IPMN with EUS-FNA vs. 22.4 months in IPMC without EUS-FNA vs. 12.5 months in PDAC, *P* = 0.64) and median OS (35.5 months in IPMN with EUS-FNA vs. 53.1 months in IPMC without EUS-FNA vs. 35.9 months in PDAC, *P* = 0.42) (Fig. 2).

During the observational period after surgical resection (24.0 months [range: 1–46] in IPMC and 17.6 months [range: 2–94] in PDAC), recurrence was observed in 58.4% of all cases (62 out of 106 cases) (Table 3). Among the 3 groups, there were no significant differences in the rates of local recurrence, distant (lung or liver) metastasis, or loco-regional lymph node metastasis. Additionally, peritoneal dissemination was observed in 25.0% of IPMC with EUS-FNA, 28.5% of IPMC without EUS-FNA and 21.4% of PDAC (*P* = 0.82).

## Discussion

In the current study, we found that the diagnostic yield of EUS-FNA performed at a pancreatic invasion site in IPMC (62.5%) was comparable to the reported sensitivity of EUS-FNA-based cytology in IPMN (64.8%) and better than the sensitivity of ERCP-based cytology (35.1%) [9]. A survival analysis and the results of long-term follow-up revealed that the procedure did not alter prognoses or the rate of recurrence, including peritoneal dissemination after surgical resection. To the best of our knowledge, this is the first study to address these issues and successfully show that this procedure provides a benefit to patients.

Although ERCP-based cytology has been utilized widely in Japan and some other Asian countries, it shows a modest diagnostic yield because the cellularity of pancreatic juice is insufficient. To overcome this low diagnostic yield, several endoscopic procedures have been developed for cytology acquisition (aspiration, brushing, and lavage) or direct biopsy with peroral pancreatoscopy. However, further studies are required to conclude which technique is optimal for IPMN with regard for technical feasibility as well as safety in clinical practice [6, 7, 11, 12]. However, EUS-FNA is widely used as a standard technique for the pathological evaluation of solid pancreatic lesions, in which it has an acceptable low incidence of adverse events [13, 14]. In this study, we applied this technique in IPMC at a pancreatic invasion site at which we could avoid puncturing cystic components of the disease. Consequently, this technique produced a better diagnostic yield than was obtained using ERCP-based cytology, as previously reported, but a much lower yield than EUS-FNA performed in solid pancreatic lesions. We speculate that one reason for this low diagnostic yield was technical restrictions related to EUS-FNA in these cases.

Regarding the safety of EUS-FNA for IPMNs, peritoneal seeding is of great concern because it can cause leakage the cyst content to leak from the lesions. However, two recent studies have revealed that preoperative EUS-FNA was not associated with an increased rate of peritoneal recurrence in patients with resected pancreatic cancer or IPMN [15, 16]. Yoon et al. included 243 patients (175 IPMNs, including 32 invasive IPMC with preoperative EUS-FNA, and 68 IPMNs, including 19 invasive IPMC without any sampling) and found that four patients (2.3%) with invasive IPMN developed peritoneal seeding in the EUS-FNA group, whereas three (4.4%, two with invasive IPMN and one with high-grade dysplasia) developed peritoneal seeding in the no sampling group (*P* = 0.403). In the current study, we focused on only invasive IPMC because it requires proper and prompt management via surgical resection. Additionally, we conducted a survival analysis to evaluate the potential harm of EUS-FNA for IPMC by comparing it to PDAC with EUS-FNA. The results showed that there were no statistically significant differences in RFS, OS and recurrence pattern between IPMC with EUS-FNA and IPMC without EUS-FNA, thus supporting the safety of EUS-FNA in pancreatic invasion sites in IPMC.

The current study has limitations stemming from the retrospective and single-center nature of the study. Therefore, the results presented here should be validated in a larger population across multiple clinical sites.

## Conclusions

Although a correct diagnosis was more difficult to obtain in IPMC than PDAC, we were able to obtain specimens without influencing the rate of recurrence or the prognosis in these patients. Hence, when a preoperative pathological evaluation is strongly recommended, EUS-FNA for pancreatic invasion sites of IPMC should be viewed as a choice to obtain specimens.

## Abbreviations

CA19–9: Cancer antigen 19–9
CEA: Carcinoembryonic antigen
ERCP: Endoscopic retrograde cholangiopancreatography
EUS-FNA: Endoscopic ultrasound-guided fine needle aspiration
IPMC: IPMN-derived invasive adenocarcinoma
IPMN: Intraductal papillary-mucinous neoplasm
OS: Overall survival
PDAC: Pancreatic ductal adenocarcinoma
RFS: Relapse-free survival
